# Previous, Current, and Future Pharmacotherapy and Diagnosis of Prostate Cancer—A Comprehensive Review

**DOI:** 10.3390/diagnostics9040161

**Published:** 2019-10-25

**Authors:** Bartosz Malinowski, Michał Wiciński, Nikola Musiała, Ilona Osowska, Mateusz Szostak

**Affiliations:** Department of Pharmacology and Therapeutics, Faculty of Medicine, Collegium Medicum in Bydgoszcz, Nicolaus Copernicus University, M. Curie 9, 85-090 Bydgoszcz, Poland; wicinski4@wp.pl (M.W.); nicole.musiala@gmail.com (N.M.); 95ilona.osowska@gmail.com (I.O.); mateuszostak@interia.pl (M.S.)

**Keywords:** prostate cancer, hormonal therapy, chemotherapy, immunotherapy, radium-223

## Abstract

Prostate cancer (PCa) is one of the most common cancers in men that usually develops slowly. Since diagnostic methods improved in the last decade and are highly precise, more cancers are diagnosed at an early stage. Active surveillance or watchful waiting are appealing approaches for men diagnosed with low-risk prostate cancer, and they are an antidote to the overtreatment problem and unnecessary biopsies. However, treatment depends on individual circumstances of a patient. Older hormonal therapies based on first generation antiandrogens and steroids were widely used in metastatic castration-resistant prostate cancer (mCRPC) patients prior to the implementation of docetaxel. Nowadays, accordingly to randomized clinical trials, abiraterone, enzalutamide, apalutamide. and docetaxel became first line agents administrated in the treatment of mCRPC. Furthermore, radium-223 is an optional therapy for bone-only metastasis patients. Sipuleucel-T demonstrated an overall survival benefit. However, other novel immunotherapeutics showed limitations in monotherapy. Possible combinations of new vaccines or immune checkpoint blockers with enzalutamide, abiraterone, radium-223, or docetaxel are the subject of ongoing rivalry regarding optimal therapy of prostate cancer.

## 1. Introduction

Cancer is the second most common cause of death in the modern world. It is estimated that in two decades the number of new cases will increase by about 70%. The most common malignancies in men are lung cancer, prostate cancer, gastric cancer, and bladder cancer. Prostate cancer (PCa) develops as a result of uncontrolled proliferation of prostate cells. In 2015, 1.6 million new cases were diagnosed worldwide, including 1.1 million in highly developed countries. Approximately 22.6% of these cases were fatal [1,2]. Prostate cancer is divided into categories based on morphology, e.g., acinar adenocarcinoma and on the stage of advancement: T—early prostate cancer located only in the prostate gland (subtypes TX, T0, T1a, T1b, T1c, T2a, T2b, T2c, T3a, T3b, T4); N—locally advanced cancer occupying adjacent lymph nodes (NX, N0, N1); M—metastatic (MX, M0, M1a, M1b, M1c) [3]. The cause of prostate cancer is still not fully understood, but there are several factors which predispose to the development of the disease: Age (the risk increases with age); ethnicity (men of Black Afro-Caribbean origin are at a higher risk than Caucasian or Asian men); genetic factors (the occurrence of prostate cancer amongst close relatives); physical activity (deficiency predisposes to the development of the disease), diet (rich in animal fats is associated with an increased risk) [2,4]. Early prostate cancer usually does not display any symptoms, and if they appear, they are similar to the symptoms associated with benign prostatic hyperplasia [5]. The treatment of advanced cancer with metastases is based on hormonotherapy which involves surgical castration, pharmacological blockage of androgen production, or blockage of testosterone from reaching cancer cells [6]. Often, despite reaching levels of testosterone akin to castration (below 50 ng/dL), progression of the disease occurs and as a result leads to the development of castration-resistant prostate cancer (CRPC) [7]. Therefore, it is of particularly high importance to find new solutions for the treatment of prostate cancer.

## 2. The Role of Androgens and Androgenic Receptors in Prostate Gland

Cholesterol derivatives belong to the steroid hormones family. Their production occurs in the interstitial cells of Leydig in men and ovaries in women, as well as in the *zona reticularis* and *zona fasciculata* of the adrenal cortex in both sexes. Androgens include: Testosterone (TTE), dihydrotestosterone (DHT)—the active form of testosterone, androstenedione, dehydroepiandrosterone (DHEA) [8]. The majority of testosterone is transported in plasma as a non-active complex with SHBG (sex hormone binding globulin) or it is bound to albumins. Around 1–2% remains free [9]. Dihydrotestosterone has the highest affinity to androgenic receptor; testosterone possesses lower affinity followed by androstenedione and dehydroepiandrosterone [8]. 

In humans, the androgen receptor (AR) is encoded by the AR gene located on the X chromosome at Xq11-12. It contains four functional domains: Transcription regulating domain, DNA binding domain, steroid binding domain, and hinge domain. The sequence found in the first of eight exons: Cytosine, adenine, guanine (CAG) seems to be associated with receptor activity [10]. In most people, the number of CAG repeats in the AR gene ranges from a few to about 36. The higher the number of repeats the lower the AR activity. However, shorter alleles may increase androgen receptor activity [10]. There were two isoforms of androgenic receptor described in 1994: AR-A and AR-B. Both isoforms are identical except that AR-A is shorter by N-terminal 187 acids compared to full length AR-B [11]. Zeng et al. [12] showed that the AR-A/AR-B ratio increased in prostate cancer. Moreover, AR-A may increase the invasion of prostate cancer PC3 cells and modulate secretion of HSP, NEP, DPM3/prostin-1 [12]. 

Available literature confirms the presence of androgen receptors in the stromal and secretory prostate cells, spermatogonies, spermatocytes, testicular Sertoli and Leydig cells, fibroblasts, sweat glands, and hairs. In addition, AR was found in the smooth muscle of the prostate, uterus, bladder, gastrointestinal tract, arteries, and arterioles [13]. 

The development and function of the prostate gland are closely related to androgen receptors (AR). ARs belong to the group of nuclear receptors and their activity is regulated by androgens: testosterone and dihydrotestosterone [14,15]. The highest affinity for the receptor is demonstrated by DHT, which is a metabolite of testosterone formed in a reaction catalyzed by the enzyme 5α-reductase in the prostate gland [16]. Activation of AR occurs through the selective attachment of one of the ligands to the Ligand Binding Domain (LBD), which results in the detachment from the receptor-heat shock protein receptor hsp90 and phosphorylation. The active hormone-receptor complex then passes through the membrane of the cell nucleus where it binds to a specific element of the hormonal response, ARE—androgen response element, in DNA and co-activators, leading to the activation of gene transcription. This mechanism is strictly controlled and, consequently, there is a balance between cell division and apoptosis [14,15]. The appearance of molecular changes results in a balance disturbance which, in turn, leads to tumorigenesis.

At present, mutations in genes and epigenetic changes which predispose to the development of hereditary or sporadic prostate cancer can be distinguished. For example, the RNASEL gene coding for Rnase L, an enzyme responsible for the induction of apoptosis and cell proliferation, has been identified within the HPC1 locus (hereditary prostate cancer 1). Familial occurrence of mutations in the L-ribonuclease gene has been observed, which in effect leads to a decrease in the enzyme activity in tumor cells [17]. Furthermore, oncogenic viruses and bacteria can directly contribute to cancer transformation. The reactive oxygen species (ROS), including superoxide, hydrogen peroxide, and nitric oxide, are released from activated phagocytes leading to DNA damage in epithelial cells. As a result, many epithelial cells become damaged. Therefore, in order to preserve the epithelial function, these cells need to be replaced by new ones which increases the risk of mutations in the DNA. Likewise, inflammatory cells secrete cytokines responsible for stimulating cell proliferation and angiogenesis processes [18].

## 3. Histology of Prostate Cancer

According to WHO, the most common histological subtype of prostate cancer is acinar adenocarcinoma which can be distinguished from several other variants: Atrophic adenocarcinoma, pseudohyperplastic adenocarcinoma, foamy gland adenocarcinoma, mucinous (colloid) adenocarcinoma, signet ring adenocarcinoma, oncocytic adenocarcinoma, and lymphoepithelioma-like adenocarcinoma [19,20]. Besides the most typical acinar adenocarcinoma, prostate cancer in 5–10% of cases may take other forms such as: Sarcomatoid carcinoma, ductal adenocarcinoma, squamous cell and adenosquamous carcinoma, urothelial carcinoma, small-cell carcinoma, basal cell carcinoma [19].

Atrophic adenocarcinoma is a variant which is characterized by cytoplasmic volume loss, infiltrative growth, the presence of macronucleoli and amphophilic cytoplasm [19,21]. Pseudohyperplastic adenocarcinoma may be localized in the peripheral zone of the gland, with papillary infoldings, cystic dilatation and also luminal undulations, branching [19,22]. Within the foamy gland adenocarcinoma cell, the cytoplasm is foamy and abundant with intracytoplasmic vesicles and pyknotic nuclei. One of the least common variants is mucinous adenocarcinoma that cell growth patterns are characterized by cribriform, tubules, cords which may line the lakes of mucin [19,20]. Signet ring carcinoma has been reported in about 60 cases. Nuclear displacement with indentation by clear cytoplasmic vacuoles and growth in small clusters can be distinguished in the signet ring cells [19,23]. Lymphoepithelioma-like adenocarcinoma is a very rare variant of prostate cancer with indistinct cell borders and syncytial growth pattern [19,24].

## 4. Diagnosis of Prostate Cancer

Prostate cancer in its early stages proceeds asymptomatically. If symptoms are present, they are often similar to benign prostatic hyperplasia (BPH). The most common symptoms are: Frequent urination, nocturia, urine hesitancy, burning or pain during urination, and slow flow. Moreover, very characteristic of prostate cancer are metastases to the lumbar spine, which can be manifested by bone pain [5]. Prostate cancer diagnosis is based on DRE (digital rectal examination) and on the determination of prostate-specific antigen (PSA) concentration in the blood serum [25]. PSA is a serine protease from the kallikrein-related peptidase family mainly produced in prostate glandular epithelial cells. Elevated levels of PSA are found in prostate cancer, but also in prostatitis and BPH [26]. For earlier detection of prostate cancer other parameters of PSA such as prostate-specific antigen density (PSAD), prostate-specific antigen velocity (PSAV), and free PSA are used. PSAD determines the ratio of concentration to the volume of the gland established on the basis of ultrasound examinations. Thus, elevated PSAD values indicate that a small volume of prostate tissue produces large amounts of PSA, while low PSAD values indicate that a large volume of prostate tissue produces small amounts of PSA [27]. A PSAV study enables the assessment of the rate of increase in PSA over time. Additionally, it must be noted that in the blood the PSA exists in a complex with alpha 1-antichymotrypsin or as a free fraction and the value of free PSA is calculated relative to the total concentration of antigen [28,29]. Furthermore, total PSA levels under 2.0 ng/mL in asymptomatic men are connected to low probability of cancer and require no further examination. PSA levels above 10 ng/mL are characteristic for patients with a high risk of cancer (biopsy is recommended). Prostate-specific antigen ratio is used to describe relative risk of cancer in the group of patients from a diagnostic “gray zone” (total PSA 4–10 ng/mL). Moreover, it is helpful in decision making which patient should undergo biopsy [30,31]. 

Approaches to perform the procedure are: Transrectal (TR) biopsy and transperineal (TP) biopsy [32]. Nowadays, transrectal ultrasound-guided (TRUS) biopsy is the most common way to collect samples. During TRUS biopsy, the needle goes through the rectal wall, then 10 to 12 samples from different areas of the prostate gland are taken [33]. However, transrectal (TR) biopsy gives false negative results of up to 49%. Moreover, risk of infection with rectal bacteria is increased due to the characteristic of the TP procedure. Therefore, fluoroquinolones are recommended [33]. In TP biopsy, the needle goes through the skin between the testicles and perineum. Transperineal approach results in lower risk of sepsis and usually requires general anaesthesia [34]. There are two possible approaches of transperineal biopsy: Template and targeted [32]. Template mapping biopsy allows to create a map of the prostate gland due to the ultrasound. A special template with holes is placed on the perineum, then needles go through it into the prostate gland [32,35]. In this method, number of cores depends on the gland volume [36]. Whereas during targeted biopsy the MRI scan is used and, as a result, fewer samples are taken. Similarities in diagnosis efficiency between TR and TP biopsy have been reported by Xiang et al. [37]. Moreover, Xiang et al. [37] recommended performing transperineal biopsy if possible.

As mentioned above, the diagnosis of prostate cancer often utilizes imaging tests: Ultrasound and magnetic resonance imaging (MRI). MRI gives a better resolution in soft tissues than ultrasound and is used to determine the place where a biopsy of a gland will be performed [38,39]. Moreover, imaging with multiparametric magnetic resonance imaging (mpMRI) may increase detection of PCa. At present, three MRI-targeted biopsy techniques have been developed: In-bore MRI-guided biopsy, MRI-US fusion software, and cognitive fusion. All aforementioned methods can be used to guide transrectal or transperineal biopsy [33]. 

Targeted biopsy (MRI-Tbx) compared to systematic biopsy gives better results in the detection of ISUP (International Society of Urological Pathologists) grade > 2. However, MRI-Tbx significantly outperforms systematic biopsy in the repeat-biopsy setting. Difference between those two biopsies becomes less significant in biopsy-naive patients. Nevertheless, MRI-Tbx compared with systematic biopsy reduces over-diagnosis of ISUP grade 1 prostate cancer. However, if mpMRI is not available, systematic biopsy is an acceptable approach. In addition, systematic biopsy combined with targeted biopsy (MRI-Tbx) may give a better prediction of the final Gleason score [25]. MRI may also allow to avoid unnecessary biopsy [37]. 

The obtained biopsies are then examined for the presence of tumor cells and evaluated according to the Gleason scale that allows for the histological assessment of tumor growth, depending on the degree of differentiation of prostate cells [39,40]. 

## 5. Prostate Cancer Therapy

In order to choose the optimal method of treatment for prostate cancer, it is necessary to consider the severity of the illness, age of the patient, and any coexisting diseases. Accordingly to current guidelines, Active Surveillance and Watchful Waiting are recommended for selected patients with localized prostate cancer. Both procedures defer invasive therapy but have a different approach. Active Surveillance is implemented in patients with a low progression risk. Those patients undergo regular controls with examination of DRE (one per year), PSA levels (every six months), MRI and biopsy (every one to three years). Invasive therapy is included when progression occurs. Watchful Waiting describes less aggressive follow-up. It does not involve regular biopsies and other frequent measurements until symptoms appear [41]. 

Surgical treatment is used in patients with non-metastatic cancer whose survival is likely to be longer than 10 years. According to SEER (Surveillance, Epidemiology, and End Results Program), in 2010–2012, 51% of men aged 18–64, 30% of men aged 65–74, and 6% of men aged 75 and over underwent radical prostatectomy (with or without radiotherapy). Isolated radiation therapy was used in 23% of men aged 18–64, 36% in men aged 65–74, and 33% in men over 75 years of age. About 20–30% of men aged 18–74 and 48% of men over 75 were not surgically treated [42]. In the case of a more aggressive course of the disease, when the patient is not eligible for radical treatment, hormone therapy (HTH) is implemented. The ADT (ADT—androgen deprivation therapy) eliminates or reduces the production of testosterone by the testicles due to the action of LHRH (luteinizing hormone-releasing hormone) agonists or LHRH antagonists [6]. Drugs that reduce androgen levels also include CYP17 (cytochrome P450-17) inhibitors (Table 1). Examples of inhibiting the action of androgens on their receptors include the use of non-steroidal antiandrogens (NSAAs) or steroid antiandrogens (SAAs) (Table 2) [43,44]. Despite many treatment methods, cancer resistance to the therapeutic regimes is becoming an increasingly common phenomenon.

LHRH agonists suppress testosterone to castration levels [49]. Overstimulation of LH release desensitizes and down regulates pituitary receptors for LHRH. Goserelin and triptorelin were accepted in the early 2000s [46,50]. LHRH agonists have shown similar effectiveness to surgical castration procedures in survival context. Therapy including LHRH agonists may be considered as a first-choice targeted treatment of prostate cancer [49]. In 2008, FDA approved Degarelix, currently the only LHRH antagonist in clinical practice [51]. Peto et al. showed that LHRH analogues therapy in localized prostate cancer may reduce deaths by one-third. The main disadvantage of LHRH therapy is a long-term toxicity problem, because castrate testosterone concentrations may decrease bone mineral density and increases the risk of myocardial infarction [52,53,54]. Moreover, the treatment affects quality of life by diminishing sexual function, increasing fatigue and risk of dementia [53,55]. 

### 5.1. Abiraterone

Abiraterone acetate is a steroidal derivative of pregnenolone. Its main pharmacologically active metabolite—abiraterone—inhibits CYP17A1 which is located in the adrenal cortex and prostate. Moreover, CYP45017A1 plays a crucial role in androgen synthesis by 17,20-lyase activity catalyzing the transformation of 17-hydroxypregnenolone to DHEA [56]. Administrated together with prednisone, it has shown a decrease in serum DHEA by 75% and testosterone to undetectable levels. Simultaneously, it was observed to reduce PSA concentration. The fact that abiraterone acetate is a strong inhibitor of CYP45017A1 is also important to mention. It is connected to its adverse effects: Hypertension and hypokalemia as the result of increased mineralocorticoids secretion [57]. These side effects may be successfully avoided by adding a low dose of prednisone (5 mg × 2 per day). It was proved by Fizazi et al. [58] in a COU-AA-301 randomized, double-blind, placebo-controlled phase III study. Moreover, abiraterone acetate improved rPFS and overall survival in patients with m-CRPC without previous chemotherapy as it was confirmed by Ryan et al. [48].

Bicalutamide, flutamide, and nilutamide belong to first generation nonsteroidal antiandrogens. Their mechanism of action is based on the competition with testosterone and other androgens for a binding place on the androgenic receptor (AR). Therefore, those drugs inhibit cell proliferation and tumor growth [66]. In 1989, Crawford et al. [67] presented LHRH and AR antagonists combined therapy which demonstrated prolongation of a patient’s life by 7.3 months in comparison to those patients who received leuprolide with placebo [68]. 

Current pharmacotherapy of CRPC accordingly to its type, includes enzalutamide, apalutamide, and darolutamide (registered on 30 July 2019). 

### 5.2. Enzalutamide

Enzalutamide is a nonsteroidal derivative of diarylothohydantoine. It has high affinity to AR and as a result inhibits the androgen binding site on the receptor (eight-fold higher than bicalutamide). Moreover, the drug not only inhibits DNA binding and AR nuclear translocation, but also induces apoptosis [68,69]. On the other hand, enzalutamide is not effective in activated AR without ligand binding domain of prostate cancer cells. The mechanism responsible for the resistance to enzalutamide is not well known yet. Korpal et al. [70] proposed the idea that the presence of resistance to enzalutamide may be associated with the mutation F876L of ligand binding domain on AR (substitution of phenylalanine to leucine—position 876). Lin et al. [71] developed an AR degradation enhancer ASC-J9, which suppresses progression of prostate cancer enzalutamide-resistant cells. It has been shown that ASC-j9 degrades androgenic receptor by intensifying its connection to murine double minute protein 2 (MDM2) [71]. However, it is still unknown if the same mechanism is responsible for the diminishing effect of AR-F877L. Bromodomain inhibitors: JQ1 and OTX015 have been proved to inhibit the resistance to enzalutamide caused by AR-V splicing [72]. 

#### 5.2.1. Safety and Efficacy Study of Enzalutamide (MDV3100) in Patients with Castration-Resistant Prostate Cancer Who Have Been Previously Treated with Docetaxel-Based Chemotherapy (AFFIRM)

In the first human study provided by Scher et al. (NCT00510718) [73], enzalutamide proved an antitumor effect in all investigated doses from 30 to 600 mg/day, and the PSA response rate reached more than 50%. However, PSA benefits were observed in a dose-dependent manner of ≤150 mg/day [73]. 

In an AFFIRM trial, 1199 patients were divided into two groups: Study (800) and placebo (399). The study group received enzalutamide capsules 160 mg/day. Patients who completed double-blind phase were involved in optional OLE Phase and underwent the same therapy until the presence of toxicity, disease progression, death, or withdrawal [62].

#### 5.2.2. Safety and Efficacy Study of Enzalutamide in Patients with Nonmetastatic Castration-Resistant Prostate Cancer nmCRPC (PROSPER)

In PROSPER, 1401 patients were divided in a 2:1 ratio to an either enzalutamide 160 mg/daily +ADT or placebo+ADT group. Primary end points included: Metastasis-free survival (described as the time from randomization to radiographic confirmed progression or death within 112 days after therapy). Secondary end points: Safety, time to PSA progression, PSA response, time of use of a new antineoplastic therapy, overall survival. Enzalutamide therapy reduced the risk of metastasis or death by 71% in comparison to placebo. The overall rate of death from any cause was lower in the study group than in the placebo group (11% vs. 13% died). Most deaths were connected to acute events that were defined by the scientists to be not correlated to the trial regimen. Adverse side effects such as: Myocardial infarction, hypertension, fatigue, and fractures were more frequent in the study group than in the placebo group [74].

### 5.3. Apalutamide

Apalutamide is a nonsteroidal antiandrogen structurally similar to enzalutamide. The mechanism of action of the drug is based on the selective binding to the LBD in AR. As a result, it prevents the binding of androgens to AR and nuclear translocation of the androgen-AR complex in target cells. This leads to the inability to bind androgen-AR complex to DNA response elements and an inhibition of gene transcription of 13 endogenous genes, e.g., PSA and TMPRSS2. It was found that apalutamide does not exhibit agonist activity at concentrations of 10 µM. Furthermore, compared to bicalutamide, it exhibits a 7- to 10-fold greater affinity for AR. Apalutamide is also an antagonist of gamma-aminobutyric acid type A (GABA_A_) receptors, but with reduced distribution in the central nervous system than other antagonists, enzalutamide [75,76].

The average oral bioavailability of the drug is 100%, and after oral administration the drug achieves the highest concentration in plasma at 2–3 h [77,78]. The metabolism of apalutamide occurs in the liver with the participation of CYP2C8 and CYP3A4 leading to the formation of the main active metabolite, N-desmethylapalutamide. Both antiandrogens are bound in the blood by plasma proteins in 96% (apalutamide) and 95% (metabolite) [78]. Apalutamide and N-desmethylapalutamide are mainly eliminated from the body with urine and, to a lesser extent, with feces [78,79]. 

It was discovered that apalutamide significantly affects the functions of cytochrome P450 enzymes. Namely, it is a strong inducer of CYP3A4, CYP2B6, a moderate inhibitor of CYP2B6 and CYP2C8, and a weak inhibitor of CYP2C9, CYP2C19, and CYP3A4. In addition, the drug may lead to the induction of UDP-glucuronosyl transferase (UGT), breast cancer resistance protein (BCRP), organic anion transporting polypeptide 1B1 (OATP1B1) and P-glycoprotein (P-gp). Therefore, apalutamide may reduce the plasma exposure to fexofenadine and rosuvastatin, which are substrates of P-gp and BCRP/OATP1B1, respectively [78].

The effectiveness of apalutamide and enzalutamide may be reduced due to a missense mutation (F876L) appearing in the LBD of the AR, which in result grants the agonist activity to the second generation NSAAs. The F876L mutation may indirectly affect the ligand-induced conformational changes of the AR binding domain in helix 12. Due to the helix 12 mediated effect on protein interactions or terminal N–C interaction, the mutation changes the dissociation of ligands and the AR response to these ligands. It was found that in equilibrium AR-binding assays, apalutamide and enzalutamide, showed a 30- and 48-fold higher affinity for the F877L mutant compared to the wild-type AR, respectively [77,80]. Mutation is detected in plasma DNA in patients with progressive CRPC after treatment with apalutamide. It is therefore believed that the F876L mutation may be a clinically important mechanism of resistance to androgens of the second generation which may adversely affect the new generation of antiandrogens [75,80]. However, it was found that effectiveness of another NSAA—darolutamide (ODM-201)—and its pharmacologically active main metabolite (ORM-15341) are not reduced in mutated variants of AR (F876L) [81].

#### A Study of Apalutamide (ARN-509) in Men with Non-Metastatic Castration-Resistant Prostate Cancer (SPARTAN) 

Castration-resistant prostate cancer (CRPC) is defined by disease progression despite ADT and testosterone levels below 50 ng/dL. If imaging studies (Technet-99m scintigraphy and computed tomography) do not indicate metastasis, this phenomenon is called castration-resistant prostate cancer. Clinically, non-metastatic castration-resistant cancer (nm-CRPC) and metastatic castration-resistant cancer (m-CRPC) are distinguished. If metastases occur (usually to bones, lymph nodes, lungs, and liver), the average life expectancy, even when using the best therapeutic methods, can be as little as three years. Clinical trials on drugs which could increase time and improve quality of life are ongoing [7,82]. 

Concomitant treatment with docetaxel and prednisone was originally used to treat m-CRPC and this regime significantly prolonged the overall survival (OS). Then cabazitaxel, sipuleucel-T, abiraterone acetate in combination with prednisone, enzalutamide, and radium-223 were introduced. In the PROSPER study in patients with nm-CRPC who continued treatment with ADT, enzalutamide have reduced the risk of nm-CRPC progression by 71% compared to ADT alone, prolonging on-metastatic survival (MFS) from 14.7 to 36.6 months [7]. 

The mechanism of action of apalutamide and enzalutamide is similar—both drugs inhibit the androgen binding to the androgen receptor. Studies on mice have shown that 3 in 19 mice displayed tumor regression with enzalutamide treatment and 13 in 20 mice with apalutamide treatment. Moreover, apalutamide can reach therapeutic levels at lower concentrations than enzalutamide. In the central nervous system, the concentration of apalutamide is twice lower than the concentration of enzalutamide, which results in a lower risk of toxicity in the nervous system. Based on the experiments on the animal models discussed above, it can be concluded that the optimal biological dose for apalutamide varies between 10–30 mg/kg/day and between 30–100 mg/kg/day for enzalutamide [75,77].

Apalutamide is currently in the third phase of clinical trials which seek to evaluate its safety and efficacy in the treatment of prostate cancer in comparison with a placebo. Within the SPARTAN study, patients were divided into two groups: The research group taking the active substance (apalutamide), and the control group taking a placebo in a 2:1 ratio. The drug is administered orally once a day at a dose of 240 mg (four 60 mg tablets) for 28 days (in a cycle). The number of cycles depends on the response of the patient’s body. Participants continue the trial until the symptoms of the disease intensify, the side effects are visible, or drug intolerance occurs [63]. 

If the dose modification did not alleviate disease progression or side effects, treatment was discontinued. The development of possible metastases was checked every 16 weeks and, if necessary, technetium-99m bone scans and CT or MRI of the pelvis, abdomen, and chest were performed. The primary end point in the SPARTAN study was metastatic-free survival. The secondary end points were: Progression symptoms, time to metastases, progression-free survival, total survival, and time to start cytotoxic chemotherapy. The HRQQL (health-related quality of life) of patients was evaluated using the Functional Assessment of Cancer Therapy-Prostate (FACT-P) and EQ-5D-3L questionnaires. Patients completed the questionnaires at baseline, on day 1 of cycle 1 (before the first dose), on day 1 of cycles 1–6, on day 1 of cycle 7, 9, 11, and 13, and on day 1 of cycle 14, 15, 16, and 17. The questionnaires were used to evaluate HRQQL changes in patients from baseline to the end of treatment. The FACT-General (FACT-G) and FACT-P results obtained at baseline were comparable to the male population standard in the USA. The study was conducted on 1207 patients (806 people in the research group, 401 in the control group). In the research group, the average PSA concentration decreased by 89.7% within 12 weeks, and in the control group it increased by 40.2%. No side effects were declared by 60–78% of patients from the research group and 64–79% of patients from the control group. A significant decrease in energy was observed in 9–17% of patients from the group obtaining apalutamide and 7–13% of patients from the control group. Metastasis developed in 30% of people. Averaged research results indicated that apalutamide did not adversely affect HRQQL (physical, social and family, emotional, and functional). A possible decline in HRQQL was caused by the disease progression occurring in both groups [63,83]. 

The most common side effects of apalutamide include: Lethargy, hypertension, rashes, diarrhea, nausea, weight loss, arthralgia, falls, hot flashes, bone injury (fracture), decreased appetite, swollen hands, ankles, or feet [78,83].

### 5.4. Darolutamide 

Darolutamide is NSAA, which mechanism of action is similar to apalutamide [64]. However, a chemical structure of darolutamide is unique and differs from other AR antagonists by consisting two pharmacologically active diastereomers [84]. Moreover, results of measurements of the inhibition constant (K_i_) and the half maximal inhibitory concentration (IC_50_) show that darolutamide compared to enzalutamide and apalutamide exhibits a greater potency, higher binding affinity (K_i_ = 11 nM; K_i_ = 86 nM; K_i_ = 93 nM, respectively), and inhibitory efficacy to AR (IC_50_ = 26 nM; IC_50_ = 219 nM; IC_50_ = 200 nM, respectively) [84]. Darolutamide appears to be less toxic than apalutamide and enzalutamide because it is characterized by negligible blood–brain barrier penetration and low binding affinity to GABA_A_ receptors [84,85]. Therefore, patients with seizures were not excluded from ARAMIS trial.

Studies on mice have shown that due to the low penetration of the blood–brain barrier, darolutamide does not increase testosterone levels in serum in contrast to enzalutamide. These results indicate a probable lack of hypothalamic-pituitary-gonadal axis stimulation by ODM-201 [84].

In contrast to other second-generation NSAAs darolutamide and its main metabolite show no clinically relevant CYP inhibition or induction at therapeutic concentrations, so the risk of interaction with other drugs is insignificant [86]. 

#### Efficacy and Safety Study of Darolutamide (ODM-201) in Men with High-Risk Non-Metastatic Castration-Resistant Prostate Cancer (ARAMIS) Trial

The clinical trial was sponsored by Orion Pharma and Bayer HealthCare. Patients included in the study had a castration-resistant prostate cancer and baseline PSA of 2 ng/mL. They were randomly assigned into two groups in the 2:1 ratio double-blind manner to receive darolutamide (600 mg in two tablets, 300 mg/tablet, twice daily) or placebo. The primary end point was metastasis-free survival (time from randomization to confirmed presence of metastasis or death from any cause). The secondary end points were overall survival and time to pain progression assessed with the BPI-SF questionnaire or opioid therapy for cancer pain, skeletal symptoms and new fractures, or tumor-related orthopedic surgical events. Trial authors investigated 1509 patients where the study group contained 955 people and 554 were present in the placebo group. The incidence of adverse events was similar in the study and placebo groups. The incidence of seizures was 0.2% in both groups. Patients with seizures were involved in trial in contrast to the PROSPER or SPARTAN trials. In ARAMIS trial, authors showed that darolutamide prolonged metastasis-free survival status by 22 months in comparison to the placebo group. Metastasis or death risk was reduced by 59% [64].

### 5.5. Radium-223

Bone metastasis is observed in almost all untreated patients. In animal studies, scientists showed the involvement of primary tumor tissue in developing bone metastasis by increasing activity of vascular endothelial growth factor (VEGF) and placental growth factor (PGF). Growth factors may create extracellular matrix to receive prostate cancer cells. Subsequently, cancer cells together with osteoblasts may induce secretion of fibroblast growth factor (FGF), transforming growth factor-beta (TGF-beta), or chemokines such as: C-C motif chemokine 22 (CCL22) or receptor activator for nuclear factor κ B ligand (RANKL) [87]. 

Radium-223 can be obtained from actinium-227/thorium-227 generator system [88]. After intravenous administration, radium-223 acts as Ca^2+^ analog and it is absorbed by bone in 25%. It is accumulated in osteoblastic activity spots. Elimination occurs in the gastrointestinal (GI) tract [89]. Radiobiological effect is associated with direct damage of tumor DNA by alpha particles. Alpha particles produce ionization around the decay site [89]. The ALSYMPCA randomized phase III trial compared radium-223 versus placebo. The study included 921 patients with CRPC and bone metastasis symptoms. All included patients showed cancer progression after docetaxel therapy or were excluded from chemotherapy. Since it has been implemented to clinical practice in 2013, over 27,000 patients underwent Xofigo therapy [90]. In 2018, the European Medicines Agency (EMA) recommended restricting the use in patients who had two previous therapies for prostate cancer with bone metastasis. Moreover, EMA suggests not to use radium-223 with other medications such as abiraterone acetate, prednisone, and prednisolone. Patients with combined therapy died 2.6 months earlier than those with a combination of placebo. Furthermore, fractures have been more frequent in patients with Xofigo (29% vs. 11% in placebo group) [91]. 

### 5.6. Chemotherapy

In 2004, the Food and Drug Administration approved docetaxel and, until 2010, it was the first line cytotoxic therapy available [92]. In 2010, cabazitaxel showed to be as effective as docetaxel. Furthermore, it can be used in docetaxel-resistant tumors [93]. Mechanism of action of both aforementioned drugs is based on their ability to secure the microtubules’ forming part of mitotic spindle which results in cell death. Immunohistochemical (IHC) analysis of samples taken from patients who underwent docetaxel therapy showed diminished AR nuclear translocation [94]. It proves that docetaxel works via androgen-depending signaling pathways [95]. Despite this, chemotherapy with docetaxel and cabazitaxel may be ineffective due to its resistance mechanism based on removal of the drug by P-glycoprotein-1. Therefore, the drug is not accumulated completely within the targeted cell and does not present cytotoxic action [96].

### 5.7. Immunotherapy

#### 5.7.1. Sipuleucel-T (Provenge)

It is the only FDA registered vaccine for the treatment of patients with asymptomatic or minimally symptomatic castration-resistant prostate cancer. The purpose of Sipuleucel-T is to stimulate the reaction of T lymphocytes against prostate cancer cells by autologous antigen-presenting cells (APCs). It is given in three injections every two weeks and has to be prepared individually for each patient. Dendritic cells are isolated from blood and then combined ex vivo with prostatic acid phosphate (PAP) and granulocyte-macrophage colony-stimulating factor (GM-CSF). The APCs present the antigens on their surface with a contribution of CD54 which is responsible for the interaction between APC and T-lymphocytes. In the next step, activated antigen-presenting cells are administrated into the patient and induce T-lymphocytes-based action against prostate cancer cells [97]. Sipuleucel-T is well tolerated and possible side effects include fever and headache (result of cytokine release). All completed phase III clinical trials showed a deferral in disease-associated pain. However, significant differences were observed only in overall survival: 25.9 months vs. 21.4 months (Clinical Trial D9901) and 19.0 months vs. 15.7 months (Clinical Trial D9902A) [98]. An Open Label, Phase II Trial of Immunotherapy with Sipuleucel-T (NCT00715104) proved that Sipuleucel-T elicited higher infiltration of CD3+ T cells into the tumor [99].

#### 5.7.2. PROSTVAC

In modern urology, in immunotherapy, viral-based vectors are used. In phase II, PROSTVAC prolonged median overall survival (OS). An average of 8.5 months was observed in comparison to placebo in mCRPC. In phase III, patients were randomly assigned into three groups: PROSTVAC group (*n* = 432), PROSTVAC with GM-CSF (*n* = 432), and placebo group (*n* = 433). Overall survival was a primary end point. Secondary end points included: Patients alive without events (AWE), pain progression, radiographic progression, a need of chemotherapy, and at-six-months death. Investigation proved clinical safety and good tolerance of PROSTVAC. However, it had no effect on overall survival (OS) or alive-without-events in mCRPC. Further experiments based on combine therapy are currently proceeding [100].

#### 5.7.3. Listeria Monocytogenes Vaccine

Currently ongoing experiments use two types of *Listeria monocytogenes* vaccine: ADXS31-142 and ADU-741. ADXS31-142 contains specially engineered truncated Listeriolysin (tLLO) which has not hemolytic domains needed for pore formation [101]. Gunn et al. [102] showed antitumor immunity induced by tLLO/tumor-associated antigens complexes in mice models. In human experiments, phase I/II open label, non-randomized clinical trial examines tolerability, and safety of ADXS31-142 alone and in combination with programmed death 1 inhibitor—pembrolizumab [103].

ADU-741 is based on a tumor-associated antigens (TAA) to ActA fusion. The precise mechanism of its action is not fully understood. ADU-741 is under investigation in a phase I clinical trial [104].

#### 5.7.4. Immune Checkpoint Inhibitors (Ipilimumab, Pembrolizumab)

Ipilimumab is a humanized monoclonal antibody IgG1κ against cytotoxic T cell antigen 4 (CTLA-4). It was approved by the FDA in 2011 for the treatment of advanced melanoma.

Results of phase III, open-label, randomized clinical trial (NCT 00861614) showed no significant difference in overall survival (OS) of patients with asymptomatic or minimally invasive mCRPC treated with combination of ipilimumab and radiotherapy. Progression free survival was improved [105].

Pembrolizumab is a humanized antibody against transmembrane glycoprotein PD-L1. PD-1 is present on T cell, while PD-L1 is expressed on antigen-presenting cells. Inhibition of PD-L1 may be a new key for cancer treatment. The results of combined therapy of pembrolizumab and olaparib, presented by Evan Yu in 2019 at Genitourinary Cancers Symposium in San Francisco are promising [106].

#### 5.7.5. CAR-T Cells in Prostate Cancer Therapy

Chimeric antigen receptor (CAR)-T cells are genetically modified T cells with expression of antigens’ chimeras and antibodies on its surface. The idea of this innovative method is to equip a patient’s T lymphocytes with an antibody-like molecule which may recognize selected antigen on the cancer cell surface and simultaneously induce cytotoxic activity of T cells [107]. Junghans et al. [108] analyzed five patients who received 109 or 1010 autologous T cells, achieving an expansion of 20–560-fold over two weeks and engraftments of 5–56%. However, unexpectedly, administrated IL-2 was depleted up to 20-fold with high engraftments of T cells. CAR-T did not show any post-treatment side effects. Two of five patients achieved clinical partial responses—PSA declines of 50% and 70% and PSA delays of 78 days and 150 days. A new pilot/phase II trial with moderate dose of IL-2 is planned [108].

## 6. New Potential Therapeutic Targets in Prostate Cancer

NF-κB and STAT3 are transcription factors which control various physiological processes such as differentiation, proliferation, and development of the cell. NF-κB and STAT3 have different signaling pathways induced by TNF-α and IL-6 [109]. Kang et al. [110] identified a number of different natural product-like compounds or metal complexes as NF-κB and STAT3 inhibitors. Benzofuran received his special attention due to its presence in natural products. Scientists conjugated benzofuran motif with Group 9 organometallic compounds (previously reported as NF-κB inhibitors). They hypothesized that a combination of benzofuran with Group 9 complexes can potentially generate dual inhibition of NF-κB and STAT3 in prostate cancer. Group 9 compounds include Complexes 1–4. Complexes 1 and 3 have iridium(III) centers while complexes 2 and 4 rhodium(III) centers. It has been found that benzofuran-conjugated iridium(III) complex 1 inhibited both IL-6-induced STAT3 activity and TNF-α-induced NF-κB-activity. Moreover, Complex 1 blocked STAT3 and NF-κB translocation from the cytoplasm to nucleus. Further examination performed on a mouse model showed promising cytotoxicity in prostate cancer cells [110]. 

In 2017, Wu et al. [111] described the role of Small Molecule Pin1 inhibitors in NF-κB signaling pathway. Prolyl-isomerase 1 (Pin1) regulates cell processes, such as cell cycle progression and apoptosis. Overexpression of Pin1 was observed in breast and prostate cancer. On the molecular level, Pin1 activates β-catenin, cyclin-D, and p65 involved in oncogenesis. Chinese scientists called their findings Compound 1. They observed that Compound 1 may disrupt Pin1–p65 interaction in PC3 cells and induce apoptosis [111].

Yang et al. [112] presented rhodium(III) Complex 1, metal-based inhibitor of lysine-specific demethylase (LSD1). Complex 1 reduced the proliferation of human prostate cancer PC3 cells and enhanced the amplification of LSD1-regulated promoters. Moreover, it did not decrease the activity of other enzymes such as monoamine oxidase (MAO) [112].

Welsh et al. [113] provided analysis of gene expression to identify new markers and/or pharmacological targets in prostate cancer. Authors of the study considered three genes as crucial: Hepsin, macrophage inhibitory cytokine-1 (MIC-1), and fatty acid synthase (FASN). Hepsin is involved in cell growth and development. Additionally, this enzyme may be a potential therapeutic target as hepsin was upregulated in all primary examined tumors. MIC-1 gene was overexpressed in 21 of the 24 tumors. Moreover, MIC-1 showed higher concentration in serum of patients with metastatic prostate, breast, and colon cancer. FASN results in dose-dependent tumor growth inhibition in a xenograft model of prostate cancer [113].

**Figure 1:**
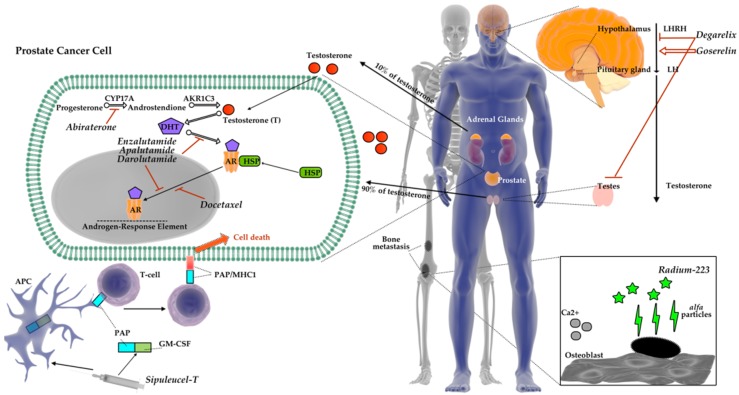
Selected drugs currently used in a prostate cancer therapy.

Mammalian target of rapamycin kinase (mTOR) occurs in two protein complexes: mTORC1 and mTORC2. mTORC1 complex via phosphorylation of ribosomal kinase S6 (S6K1) and Eukaryotic Translation Initiation Factor 4E-binding protein 1 (4E-BP1) initiates translation of crucial proteins involved in cell cycle progression. Ding et al. [114] found that 4E-BP1 and 4E-BP2 may slow down a tumor growth in prostate cancer via mTOR activation due to PTEN (phosphatase and tensin homolog deleted on chromosome ten) loss. Authors suggested that mTOR inhibitors can be used alone or in combination. However, the main disadvantage of above-mentioned treatment is that it may promote the survival of hypoxic cells resistant to other forms of the therapy [114]. Figure 1 shows currently available drugs used in prostate cancer therapy.

## 7. Conclusions

Prostate cancer is one of the most common malignancies in men that, in early stages, is often asymptomatic. However, if symptoms are present, they resemble the one observed in BPH. Current diagnosis of prostate cancer includes rectal examination, PSA tests, imaging tests, and biopsy. It is of great importance to determine the severity of prostate cancer, assess the risk, coexisting diseases, and the age of the patient in order to choose the optimal treatment. Active surveillance and/or watchful waiting are recommended for patients with asymptomatic or slowly-growing cancer. Surgical treatment may be used in the case of non-metastatic PCa. However, in advanced metastatic PCa, the treatment is based on hormone therapy. There is no first-choice hormonal therapy for metastatic prostate cancer, but for patients with impending spinal cord compression LHRH antagonists are preferred. Combination therapy of LHRH with NSAA (i.e., flutamide) for complete androgen blockade shows small survival advantage (<5% vs. monotherapy beyond five years of survival) and it is associated with increased side effects and long-term toxicity problems. 

Combination of abiraterone acetate (1000 mg/day), prednisone (5 mg/day), and ADT shows significant benefits in overall survival (38% at three years) and secondary end points. It should be considered as a standard to avoid possible mineralocorticoid-related side effects (especially in hormone-sensitive metastatic prostate cancer). 

In metastatic patients, docetaxel (75 mg/sqm) and ADT can be used. However, all chemo-hormonal clinical trials presented hematological side effects: 12–15% Grade 3–4 neutropenia and 6–12% Grade 3–4 febrile neutropenia. Therefore, granulocyte colony-stimulating factor receptor (GCSF) is recommended to reduce above-mentioned toxicity. 

Due to the frequent development of resistance to the therapeutic regimens, there is a need to introduce new drugs. Second-generation NSAAs (enzalutamide, apalutamide, darolutamide) prolong metastasis-free survival and time of progression of patients with m-CRPC. Enzalutamide not only presents benefits in rPFS (*p* < 0.0001) and OS (*p* < 0.0001), but is also well tolerated in patients above 75 years. However, secondary diseases and liver condition should be taken into consideration before NSAAs implementation as patients with liver metastasis showed no benefits from enzalutamide therapy. It emerged that apalutamide (compared to enzalutamide) can reach therapeutic levels at lower doses which may result in a reduced risk of toxicity. Currently, apalutamide is in the third phase of the clinical trials. Nevertheless, it must be considered that the efficacy of enzalutamide and apalutamide may be reduced in patients with a missense mutation (F876L). It was found that the F876L mutation that appears in the LBD of the androgen receptor may cause to confer agonist activity on enzalutamide and apalutamide but not on darolutamide. In addition, darolutamide has low blood–brain barrier penetration; therefore, it is not associated with seizures. Darolutamide has been approved by the FDA in August, 2019.

For patients with mCRPC who eventually progress, cabazitaxel, abiraterone after prior docetaxel, enzalutamide after docetaxel or radium-223 should be considered as second-line therapy. It is recommended to administrate 20 mg/sqm instead of 25 mg/sqm of cabazitaxel as it shows lower toxicity and similar effectiveness. Cabazitaxel therapy should include GCSF prophylaxis to avoid neutropenia. 

Radium-223 may be administrated to patients with bone metastasis in spite of undergoing two previous therapies for PCa. Six injections of 50 kBq/kg radium-223 improved median OS by 3.6 months. However, one should remember not to use radium-223 with other drugs, e.g., abiraterone acetate.

Sipuleucel-T might be a good alternative for asymptomatic or minimally symptomatic metastatic castration-resistant patients, but currently it is not available in Europe. Nonetheless, clinical trials are currently underway on another immunotherapy—vaccine PROSTVAC, although phase III findings do not support positive outcomes from phase II. *Listeria monocytogenes* vaccine undergoes phase I/II as monotherapy and as combination with pembrolizumab. However, the results derive from study performed on a small group of patient (*n* = 50). 

CAR-T cells have shown promising results in animal studies, but their safety and effectiveness need further investigation in clinical trials scale investigation. CAR-T-depending signaling pathways as well as the role of stem cells in CAR-T therapies remain unknown.

Immune checkpoint inhibitors are promising in combined therapy with olaparib and pembrolizumab. However, available literature shows that only part of the patients might benefit from this therapy. Moreover, further investigations are necessary to properly identify biomarkers that may determine inclusion criteria for those patients.

In summary, due to the emerging resistance of prostate cancer cells, it is of great importance to continue the research for new therapeutic solutions in the treatment of PCa and to properly use currently existing therapies.

## Figures and Tables

**Table 1:** Examples of drugs to reduce levels of androgens.

Drug Classification	Example of Drug	Mechanism of Action	Clinical End Point
LHRH agonists	Goserelin Triptorelin	Induces testosterone suppression by binding to LHRH receptors in pituitary gland; thus, acting as an agonist that stimulates the production of LH and FSH. As a result, it promotes the production of testosterone in a non-physiological way. Finally, TTE levels decrease due to the regulation of the hormonal feedback systems.	Clinical trials have shown a reduction in serum testosterone levels below the castration level [45,46].
LHRH antagonists	Degarelix	Induces testosterone suppression by binding to LHRH receptors in pituitary gland; thus, blocking their interaction with LHRH and then reducing the level of LH and FSH.	Treatment resulted in suppression of testosterone levels to ≤0.5 ng/mL between 28 and 364 days [47].
CYP17 inhibitor	Abiraterone acetate	Lowers androgens (DHEA, TTE, DHT) levels by irreversible inhibition of CYP17A1 activity. Furthermore, it has partial affinity to AR.	Treatment resulted in 57% prolonged radiographic progression-free survival (rPFS) and 25% decrease in the risk of death [48].

**Table 2:** Examples of antiandrogenic drugs.

Drug Classification	Example of Drug	Mechanism of Action	Clinical End Point
First-generation NSAAs	Flutamide	The mechanism is based on the selective binding of the drug to the AR, which blocks the effects of androgens (TTE, DHT) on prostate cells.	Flutamide with LHRH-A prolong the survival and time to progression [59].
	Nilutamide		Nilutamide with orchiectomy improve survival and prolong time to progression [60].
	Bicalutamide		Bicalutamide with LHRH-A lower TTE levels and prolong the median survival compared with the flutamide with LHRH-A combination [61].
Second-generation NSAAs	Enzalutamide		Prolongs the survival of patients with m-CRPC after chemotherapy [62].
	Apalutamide		Prolongs metastasis-free survival and time to progression of patients with nm-CRPC [63].
	Darolutamide		Prolongs metastasis-free survival of patients with nm-CRPC [64].
SAAs	Cyproterone acetate	SAAs act similarly to NSAAs, but in addition to blocking the effects of androgens, they also suppress their gonadal production.	SAAs exhibit poor effectiveness in prostate cancer therapy (lower than NSAAs) and have more side effects [65].

## Abbreviations

ADT	androgen deprivation therapy
AR	androgen receptor
ARE	androgen receptor element
BPH	benign prostatic hyperplasia
CRPC	castration-resistant prostate cancer
CYP17	cytochrome P450-17
DHEA	dehydroepiandrosterone
DHT	dihydrotestosterone
GABA_A_	gamma-aminobutyric acid type A
LBD	ligand binding domain
LHRH	luteinizing hormone-releasing hormone
m-CRPC	metastatic castration-resistant prostate cancer
nm-CRPC	non-metastatic castration-resistant prostate cancer
NSAA	non-steroidal antiandrogen
PCa	prostate cancer
PSA	Prostate specific antigen
PSAD	prostate-specific antigen density
PSAV	prostate-specific antigen velocity
SAA	steroid antiandrogen
